# Supplementary material for: Heat adaptation of phage T7 under an extended genetic code
Source: Virus Evol. 2021 Dec 1;7(2):veab100. doi: 10.1093/ve/veab100 (PMC8923235; doi:10.1093/ve/veab100)
Supplement: veab100_Supp [file veab100_supp.zip › Sequencing_Analysis.zip!/Sequencing_Analysis/A3/indexA3.html]

BRESEQ :: Mutation Predictions


|  |  |
| --- | --- |
|  | ***breseq***  version 0.30.1    mutation predictions \| marginal predictions \| summary statistics \| genome diff \| command line log |

| Predicted mutations | | | | | |
| --- | --- | --- | --- | --- | --- |
| evidence | position | mutation | annotation | gene | description |
| MC JC | 1,253 | Δ1,489 bp | intergenic (–/–) | – / – | –/– |
| MC JC | 13,278 | Δ1,019 bp | intergenic (–/–) | – / – | –/– |
| RA | 15,094 | G→T | intergenic (–/–) | – / – | –/– |
| RA | 20,544 | G→A | intergenic (–/–) | – / – | –/– |
| RA | 21,265 | A→C | intergenic (–/–) | – / – | –/– |
| RA | 24,161 | A→C | intergenic (–/–) | – / – | –/– |
| RA | 24,238 | A→G | intergenic (–/–) | – / – | –/– |
| RA | 24,475 | A→G | intergenic (–/–) | – / – | –/– |
| RA | 26,746 | G→T | intergenic (–/–) | – / – | –/– |
| RA | 27,783 | G→A | intergenic (–/–) | – / – | –/– |
| RA | 27,932 | T→C | intergenic (–/–) | – / – | –/– |
| RA | 28,692 | A→G | intergenic (–/–) | – / – | –/– |
| RA | 29,258 | A→G | intergenic (–/–) | – / – | –/– |
| RA | 35,095 | A→G | intergenic (–/–) | – / – | –/– |
| RA | 35,366 | C→A | intergenic (–/–) | – / – | –/– |
| RA | 37,346 | T→C | intergenic (–/–) | – / – | –/– |

| Unassigned missing coverage evidence | | | | | | | | | | |
| --- | --- | --- | --- | --- | --- | --- | --- | --- | --- | --- |
|  |  |  | seq id | start | end | size | ←reads | reads→ | gene | description |
| \* | \* | ÷ | NC\_001604.1 | 1 | 110 | 110 | NA [0] | [1055] 1062 | *–/–* | –/– |

| Unassigned new junction evidence | | | | | | | | | | | |
| --- | --- | --- | --- | --- | --- | --- | --- | --- | --- | --- | --- |
|  | | seq id | position | reads (cov) | reads (cov) | score | skew | freq | annotation | gene | product |
| \* | ? | NC\_001604.1 | = 19635 | 393 (0.230) | 1117 (0.650) | 309/590 | 0.2 | 74.6% | intergenic (–/–) | *–/–* | –/– |
|  | ? | NC\_001604.1 | 19645 = | 374 (0.220) |  |  |  |  | intergenic (–/–) | *–/–* | –/– |
